# ‘We do not do any activity until there is an outbreak’: barriers to disease prevention and health promotion at the community level in Kongwa District, Tanzania

**DOI:** 10.3402/gha.v7.23878

**Published:** 2014-07-30

**Authors:** Tumaini Nyamhanga, Gasto Frumence, Mughwira Mwangu, Anna-Karin Hurtig

**Affiliations:** 1 Department of Development Studies, School of Public Health and Social Sciences, Muhimbili University of Health and Allied Sciences, Dar es Salaam, Tanzania; 2 Epidemiology and Global Health, Umeå International School of Public Health, Umeå University, Umeå, Sweden

**Keywords:** barriers, disease prevention, health promotion, Tanzania

## Abstract

**Background:**

Little is known about the barriers to disease prevention and health promotion at the community level – within a decentralized health system.

**Objective:**

This paper, therefore, presents and discusses findings on barriers (and opportunities) for instituting disease prevention and health promotion activities.

**Design:**

The study was conducted in Kongwa District, Tanzania, using an explorative case study approach. Data were collected through document reviews and in-depth interviews with key informants at district, ward, and village levels. A thematic approach was used in the analysis of the data.

**Results:**

This study has identified several barriers, namely decision-makers at the national and district levels lack the necessary political will in prioritizing prevention and health promotion; the gravity of prevention and health promotion stated in the national health policy is not reflected in the district health plans; gross underfunding of community-level disease prevention and health promotion activities; and limited community participation.

**Conclusion:**

In this era, when Tanzania is burdened with both communicable and non-communicable diseases, prevention and health promotion should be at the top of the health care agenda. Despite operating in a neoliberal climate, a stronger role of the state is called for. Accordingly, the government should prioritize higher health-protecting physical, social, and economic environments. This will require a national health promotion policy that will clearly chart out how multisectoral collaboration can be put into practice.

The World Health Organization (WHO) and ministries of health in low- and middle-income countries have recently – albeit rhetorically – sounded a need to revitalize the primary health care (PHC) approach for health improvement (1). This can partly be attributed to the challenge of the double burden of disease experienced by low-income countries (2). That is, non-communicable diseases (NCDs) – for example, heart disease, cancer, and diabetes – are increasingly becoming a cause of morbidity and mortality in poor countries, while communicable diseases remain highly prevalent and are also predisposing some NCDs.

The impact will be particularly felt in sub-Saharan Africa, where NCDs will account for 46% of all deaths by 2030, up from 28% in 2008, and in South Asia, which will see the share of deaths from NCDs increase from 51 to 72% during the same period (3). These poor regions of the world will, at the same time, continue to grapple with the widespread prevalence of communicable diseases such as HIV, malaria, tuberculosis, and mother and child conditions, and so face a ‘double burden’ of disease not experienced by their wealthier counterparts.

The magnitude of the challenge notwithstanding, there is hope for turning the tide if prevention and health promotion at the community level are made a priority (4–7), as advocated in the PHC approach. According to WHO, PHC refers to ‘essential health care based on practical, scientifically sound, and socially acceptable method and technology; universally accessible to all in the community through their full participation; at an affordable cost; and geared toward self-reliance and self-determination’ (8). It is a comprehensive approach to service provision that involves activities targeting the individual (curative services) and the general population (health promotion and disease prevention). From the above-cited definition, two core values – universal health care (social justice) and community participation – guide PHC (9). As such, success of PHC is determined by collaboration between health sector and related sectors such as water, agriculture, education, finance, and communication. The collaboration should be people-centered – that is, community members should be involved in agenda setting, analyzing problems, planning, and implementation of interventions (10).

Tanzania's health policy (11) categorically states that PHC remains the cornerstone in the delivery of essential health care in the country. PHC as a value-based systems approach for all levels (Alma Ata 1978) thus still remains a point of reference that is emphasized by the government in the National Health Policy, as indicated in Box 1.

Box 1 **Primary health care**

In its endeavor to ensure success in delivery of essential health care in the country, the Government [of Tanzania] through Primary Health Care emphasizes on:

- Community involvement and ownership through active participation in identification of problem areas, planning, implementation, monitoring, and evaluation of health care services;
- Multisectoral collaboration by establishment of committees involving other sectors such as water, agriculture, and education, and ministries such as community development, gender, and children;
- Equity and accessibility to health care by ensuring that every individual has the right to health care and equitable distribution of health resources in the country;
- Empowerment through decentralization of health services to regions and districts and communities to ensure effective coordination, implementation, supervision, and provision of quality health care to the community;
- Providing promotive, preventive, curative, and rehabilitative interventions to all individuals and families with their active participation.

The health policy also states that health sector reforms seek to operationalize the same basic principles of PHC in the provision of health care services. The response in most low-income countries like Tanzania was the development of essential health care package which is an integrated collection of cost-effective interventions. The package comprises the following: reproductive and child health services, control of communicable and NCDs, treatment of common conditions of local prevalence within the district, community health promotion and disease prevention through environmental sanitation, and management and occupational health services.

Although Tanzania's Health policy (11) theoretically embraces the above-described principles of PHC – which are key to the health for all strategy – there has not been sufficient conformity with these principles to bring about the desired improvement in people's lives. This may partly be a result of limited intersectoral collaboration for health development and limited community involvement. This deficiency is evidenced by poor development indicators such as low life expectancy and high child and maternal morbidity and mortality (2).

## PHC in Tanzania: before the Alma-Ata declaration

Tanzania started implementing the principles of PHC long before the 1978 Alma-Ata Declaration (12). Right after independence, the first president of Tanzania, Mwalimu Julius Kambarage Nyerere, issued a philosophical statement saying that Tanzania (then Tanganyika) had three enemies that she had to fight: poverty, illiteracy, and ill health (13). This philosophical commitment was operationalized by being integrated into the national policy popularly known as the Arusha Declaration of 1967 (14). One of the aims of the Arusha Declaration was that government should exercise effective control over the principal means of production and pursue policies that facilitate collective beneficence from national resources. The policy thus ordered free provision of social services, notably health, education, and water (14). It is worth noting that the implementation of the Arusha Declaration marked a shift in the focus of service delivery from a predominantly curative to a more comprehensive PHC approach. That is, more resources were directed toward training middle- and lower-level cadres of health workers, and the construction of health facilities in rural settings (dispensaries and health centers). In addition, community health programs on nutrition, literacy, and sanitation received both financial and political support.

However, a gradual shift away from investing in prevention and health promotion started in the 1990s when Tanzania embarked on neoliberal-oriented health sector reforms. These reforms were preceded by the economic crisis of the late 1970s and early 1980s that necessitated Tanzania, like many African countries, to undertake socioeconomic reforms (15). The reforms involved the adoption and implementation of structural adjustment programs (SAPs) prescribed by the International Monetary Fund and the World Bank (16). The SAPs specifically involved implementation of the following measures: removal of subsidies; budget; liberalization; privatization; currency devaluation; and cuts in social services (16).

## Health sector reforms and PHC

Health sector reforms are defined as intensive long-term efforts to strengthen and improve health systems and, ultimately, improve the nation's health (17, 18). Health sector reforms are aimed at increasing the effectiveness, efficiency, quality, equity, and financial soundness of health systems (19). In Tanzania, as in other developing countries, the reforms were prompted by a number of factors, including the movement from a state-controlled economy to market-oriented economies; insufficient funding for health; and the poor quality, low accountability, and inefficiency of existing health services (20). The reforms measures can be grouped into three broad categories: ideological and policy changes, organizational changes, and financing changes.

Ideological reforms involved reducing the role of the state from being the sole provider of health services to that of being a partner and a creator of a conducive environment for other actors – private, civil society, and non-governmental organizations (NGOs) (21). More importantly, in the Tanzanian context, the ideological reform involved disposing of the egalitarian policy of free provision of health services put in place by the Arusha Declaration (22).

Organizational reforms entailed decentralization, which involved transfer of power, authority, and functions from the central to local government authorities (23). Decentralization took place in three areas: fiscal, political, and managerial/administrative. Fiscal decentralization involved allowing local authorities to collect taxes from their own sources for covering recurrent expenditures (4). On the contrary, the central government retained the authority to mobilize and disburse health block grants and basket funds. Health block grants come from the central government's internal revenues and are spent on personnel emoluments, other charges, and a development grant, whereas basket funds are finances that are contributed to the country by several bilateral and multilateral development partners and spent on other charges (24, 25). Moreover, political decentralization involved the transfer of political authority and function from central government ministries to the local government authorities – cities, municipalities, and district councils (26). The Prime Minister's office – Regional Administration and Local Government and Health ministries – and the Ministry of Health and Social Welfare retained the authority for formulating policies, guidelines, standards, and regulation of health practice. However, the local government authorities were entrusted with the responsibility of planning and utilizing available resources to meet health needs in their areas of jurisdiction (27). Reports on evaluation of PHC show that many countries that have been implementing health sector reforms adopted selective PHC with curative care and few aspects of disease prevention such as vaccination and encouragement of long-term breastfeeding (9).

Little is known about local realities regarding the impact that health sector reforms have had on broader prevention and health promotion programs. The aim of this paper, therefore, is to shed light on barriers to implementation of comprehensive primary health – specifically disease prevention and health promotion activities at the community level in the context of health sector reforms.

## Conceptual framework

**Fig. 1:**
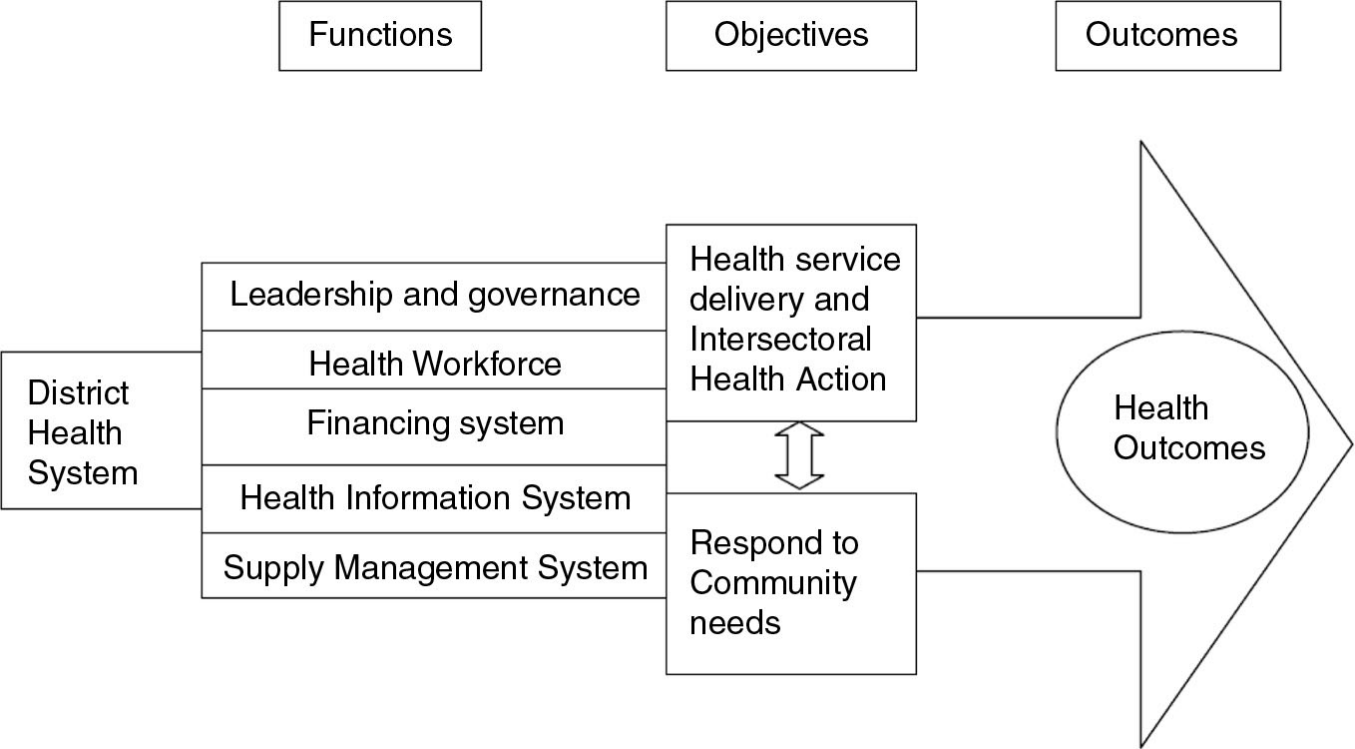
Conceptual Framework. *Source*: Adapted from Saachy et al. (29).

This paper is guided by the WHO's system framework (28) as interpreted from the perspective of PHC by Saachy et al. (29) (Fig. 1). Although the original adaptation by Saachy et al. focuses on the national health system, the current study applies the framework at both national and district levels. This is because the two levels of governance work interdependently. Whereas the central (national level) government develops a health policy and allocates resources, the local (district level) government is entrusted with a duty of implementing the policy.

**Fig. 2:**
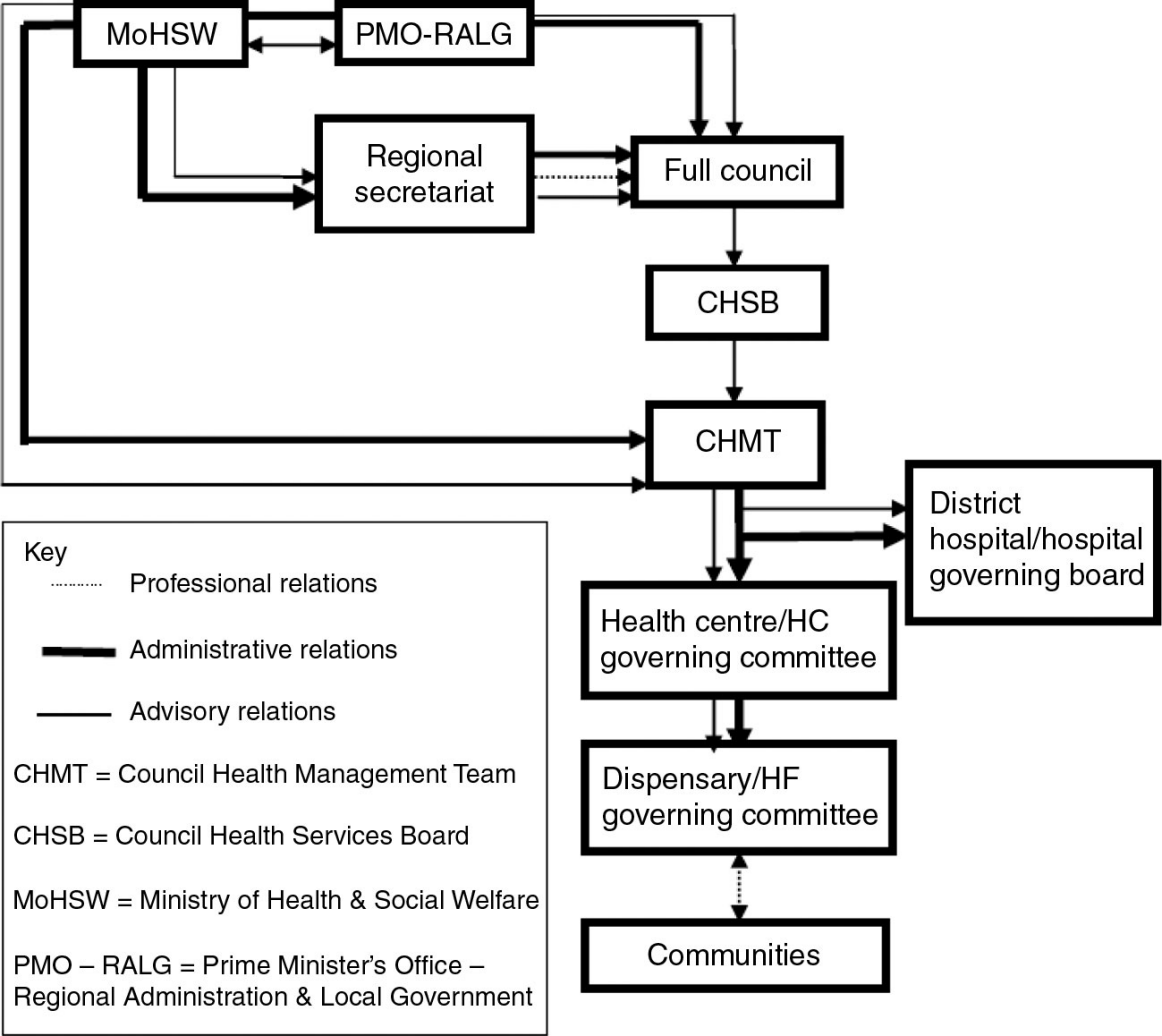
Organization structure of a decentralized health system. *Source*: Modified from the figure on interlinks between central and local government structures (Molel, 2010).

Thus, in the context of Tanzania, the objectives of the district health system are to deliver accessible, equitable, and good-quality health services that are both responsive to community demands and based on the principle of inter-sectoral collaboration.

The framework illustrates that for the district health system to attain the set objectives there are specific functions that must be fulfilled. These functions include the development of human resources, a health information system, service delivery, financing, and governance. The current study will focus on the latter three functions.

In Tanzania, there are well-established structures at the national, district, and community levels for managing the above-mentioned functions of the health system (Fig. 2). The core structure at the district level is the council health management team (CHMT), which carries out the following key functions: planning, coordination, implementation, monitoring, and evaluation of all health activities. In essence, the CHMT oversees service delivery at the hospital, health center, dispensary, and community levels (23). The communities are linked to the health system through health facility governing committees, which have been formed by a local government act (30). It was envisaged that the committees would assist health center and dispensary management teams in planning and supervising community health programs (31) and that they would facilitate the empowerment of households and communities with the knowledge and skills needed to reduce the burden of disease (21). In addition, there are government structures at ward and village levels – ward development and village health committees – that play a role in providing political leadership and enhancing inter-sectoral linkages for health development in the communities they serve.

## Methods

### Study setting

This study was conducted from January to February, 2013, in Kongwa District. Kongwa is a site for the implementation of a collaborative health system research project between Muhimbili University of Health and Allied Sciences in Tanzania and Umea University in Sweden. The choice of Kongwa was purposively made to represent a typical rural district that has a moderate level of socioeconomic development and is fairly accessible in terms of transportation and communication networks.

Kongwa is one of the districts in Dodoma region. It is bordered by the Manyara region to the northern part, to the east by the Morogoro region, to the south by the Mpwapwa district, and to the west by the Dodoma rural district. Administratively, the district has 14 wards and, according to the 2002 Tanzania National Census, the population of Kongwa District was 249,760, with an annual growth rate of 2.4% (12). The health care system in Kongwa District is largely based on public health facilities, including one district hospital, three health centers, and 27 dispensaries.

### Study design

This paper is based on an explorative case study. Qualitative case study methodology enables researchers to explore and explain a phenomenon within its context using multiple sources of data (32). The approach was relevant to this study because it involved in-depth investigation of barriers and opportunities for instituting disease prevention and health promotion activities at the community level in the context of health sector reforms in the study district.

### Study population and their sampling criteria

The recruitment of the study participants in this sub-study was *purposive*, as it took account of their positions and experience in dealing with prevention and health promotion at the community level. Table 1 summarizes the details of the study respondents.

**Table 1:** Study respondents

S/N	Category	Number	

		Male	Female
1	CHMT members	5	2
2	Other key district officials (DPLO, DT, CCHS)	3	1
3	Heads of programs	2	2
4	Ward executive officers	2	0
5	Members of Village health committee	1	1
6	Members of health facility governing committee	2	1
6	Ward health officer	1	0
	Total	16	7

As Table 1 shows, 23 respondents were involved in the study. Out of these, there were seven CHMT members, four key district officials (District Planning Officer, District Treasurer, Chair of Council Health Services); four heads of programs (RCH Coordinator, District AIDS Coordinator, Malaria Coordinator, and TB/Leprosy Coordinator); two Ward Executive Officers; one Ward Health Officer, five members of the Village Health Committee; and three members of the Health Facility Governing Committee.

### Data collection methods

The data were collected through conversation by face-to-face in-depth key informant interviews with the officers concerned, when they could find a convenient time. In addition, telephone interviews were also undertaken when further clarification was sought on data collected face-to-face. An interview guide with a list of open-ended questions was used. These questions covered major relevant areas such as: priority in allocation of resources – prevention versus curative care; planning for prevention and health promotion activities at community level; community participation; and local leadership structures for prevention and health promotion.

Additionally, several supporting documents that were cited by the respondents, and others that were deemed relevant by the investigators, were reviewed. These included: the National Health Policy of 2007 and the council's comprehensive health plan (CCHP) for Kongwa. These documents were read with a view of understanding articulation of and efforts put on health promotion and disease prevention activities at the community level.

### Data processing and analysis

Tape-recorded in-depth interviews were transcribed verbatim, translated from Kiswahili into English. Supplementary field notes were typed immediately after data collection, using a word processor. Additional information that was obtained later on the phone was also included. A thematic analysis approach was used in analyzing the study's findings through the following steps (33). First, transcripts and field notes were read several times in order to identify patterns of experience (categories of common ideas). Second, all data that relate to the already classified patterns were identified and placed within the corresponding pattern. Third, patterns of experience were examined to determine similarities and differences among them with respect to the meanings they conveyed in relation to the research question. Fourth, the patterns of experience were combined into study themes that represented a higher level of patterned response or meaning within the data set (34). These study themes are highlighted in the introductory part of the results section.

### Trustworthiness

The trustworthiness of the findings can be secured through different approaches such as: triangulation, prolonged engagement, peer-debriefing, and member checks (35). In this study, key informant interviews were complemented with document reviews to triangulate sources of information. The interview guide was piloted to improve the questions and the moderating skills. The data collection period was prolonged to allow the researchers enough time for reflection between field visits and to allow them to perform a preliminary analysis that guided their subsequent data collection. The final data analysis was facilitated by peer-debriefing sessions involving a multidisciplinary research team.

### Ethical considerations

The study obtained ethical clearance from Muhimbili University of Health and Allied Sciences. The respondents were requested to participate in the study, and provided their oral consent. Prior to consenting, they were assured of confidentiality by being told that no names were required and that no unique identifiers would feature in the study report or on publication. Furthermore, each potential respondent was informed of his/her right to decline outright to participate, or to withdraw consent at any stage of the interview, without undesirable consequences.

## Results

In this section, we present results on barriers to implementation of disease prevention and health promotion activities at the community level in the context of health sector reforms. The results are organized around the study themes that reflect the three elements of the district health system in the conceptual framework, namely: service delivery, financing, and governance. It is worth noting that there is an inevitable overlap that shows a close interdependence among the constituent elements of the health system. The study themes are lack of political will toward delivery of comprehensive PHC; underfunding of prevention and health promotion; Ill-preparedness of the ward and village authorities; and limited community participation.

### Lack of political will toward the delivery of comprehensive PHC

The readiness for implementing comprehensive PHC was assessed through key informant interviews and a review of important documents – the National Health Policy of 2007 and the Council Comprehensive Health Plan. The respondents indicated that both national- and district-level decision-makers lack political will in prioritizing prevention and health promotion activities. Their main focus is on the curative aspect of health care. When asked why prevention and health promotion activities were not a top priority, one of the key informants, a member of the CHMT said:

I think maybe it is the system from the ministry down to the district level, as I do remember sometimes I went to the ministry for budgeting, and someone at the ministry commented that why don't we remove the activity of health promotion and instead put procurement of medicines? You know, in Tanzania, we are unlike the developed countries. Here curative care takes precedence over preventive, while in other countries it is vice versa, so we have to focus now on preventive rather than curative care. (KI-1, Kongwa)

Another member of the CHMT added that:

Sometimes during budgeting someone at the Ministry commented that we as the Ministry [government] have decided to put our efforts into medicine so remove this item [health promotion]. So you find that we planned for preventive cure from district level or lower level but at the end of the day the planning process is not participatory; the Ministry dictates which items should be funded and which ones shouldn't. It is the directive from the Ministry that matters. (KI-4, Kongwa)

In addition, a review of documents shows that although the National Health Policy nicely explains the importance of prevention and health promotion, translation of the same into a district health plan is a major challenge. Prevention and health promotion activities are not well articulated in the comprehensive district health plan. As Table 2 shows, prevention and health promotion activities are scarcely indicated. For instance, for reducing mortality due to malaria the plan contained only one preventive activity: to provide a *hati punguzo* (a voucher for accessing a subsidized mosquito net) for all pregnant women attending ANC by 100%. The table also shows that preventive activities for diarrhea are missing – suggesting a neglect of hygiene, water supply, and sanitation. Likewise, activities for addressing NCDs are sparingly indicated. For instance, in the 2011 comprehensive health plan, control of NCDs is missing from the community cost center. It is only indicated under the health center cost center, where it is shown that screening for cervical cancer will be conducted.

**Table 2:** Objectives, targets, and planned interventions for selected diseases – 2010/11

Problem	Objective	Target	Activity
High morbidity and mortality rate due to malaria	To reduce morbidity and mortality due to malaria by 50% by year, 2015 To reduce morbidity and mortality due to malaria by 7% by June 2011	1) Training to 20 health workers on malaria case mgt. 2) Commemoration of African Malaria day. 3) Computer training to malaria/IMCI data manager. To provide HATI PUNGUZO for all pregnant woman attending ANC by 100%.	1) Training to 20 health workers on malaria case management. 2) Commemoration of African Malaria day. 3) Computer training to malaria/IMCI data manager. To provide HATI PUNGUZO for all pregnant woman attending ANC by 100%.
High morbidity and mortality rate due to communicable diseases such as, Diarrhea Tuberculosis and HIV/AID/STI	To increase proportion of TB cases completed treatment from 97 to 100% by June 2009 To reduce STI/HIV/AID cases	To increase case mgt ability and case detection in all 41 HF. Reduce HIV infection from 3.1 to 2.0%.	To train 20 clinician/nurses on proper case management for 4 days. Tracing of defaulters Conduct supportive supervision to 41 health facilities. 1) Training 20 health providers on proper management of STI. 2) Training of 20 COs on syndomic case management. To conduct supportive supervision on syndomic case management to 41 health facility.

*Source*: Kongwa Comprehensive Council Health Plan for the Year 2010/2011.

Nevertheless, the CCHP shows a window of opportunity for addressing environmental sanitation. There is a statement that the council would facilitate the private sector to invest in environmental health activities. It reads: ‘The council will facilitate private sector and civil society organizations to invest in the collection and disposal of refuse’ (2010/2011 CCHP, Kongwa).

### Underfunding of prevention and health promotion

Document reviews show that prevention and health promotion activities are grossly underfunded. From the resource allocation point of view, such activities are marginalized as only 2–5% of district health funds are allocated for them, as indicated in Table 3.

**Table 3:** Allocation ceiling for the health basket fund and block grant (other charges)

Cost center	Ceiling range for allocation by council (%)
Office of District Medical Officer (DMO)	15–20
Council hospital/CDH	25–30
Voluntary agency hospitals (VAH)	10–15 (health basket funds only)
Health centers	15–20
Dispensaries	20–25
Community initiatives	2–5

*Source*: URT (24).

Additional evidence of under-funding is indicated in Table 4, which shows that money budgeted under community health initiatives and promotion to a large extent targets two main areas. One, meeting travel costs and subsistence allowance for the district health staff who carry out outreach visits. Two, waste disposal and water availability in the health facilities. The table makes no mention of support for waste disposal, sanitation, and/or water availability at the household level. As a consequence, there is limited community participation in utilizing these funds. It was reported that there are no government funded community activities initiated and implemented by local people themselves.

**Table 4:** Performance budget framework for 2012/13 – Cost Centre E07 Community Health Initiatives/Promotion

Intervention	Activity	Details				Output indicator

		Description	Unit	Qty	Unit cost	
Case management (diagnosis and treatment) of locally important	To conduct NTDs Outreach at community and conduct BTRP Surgeries BY June 2013	Petrol	Liters	1	600,000.00	Number of outreach conducted
diseases and neglected tropical diseases		Diesel	Liters	776.0	2,500.00	
		Per diem – domestic	Person	1.0	1,950,000.00	
Promotion and prevention predisposing factors for NTDs at community level	To sensitize community on NTDs through commemoration of vision world sight meetings by June 2013	Per diem – domestic	Person	1.0	195,000.00	Activity report presented
		Ground travel (Bus, Railway, Taxi)	Liters	1.0	20,000.00	
Mobilized adequate funding for health and social welfare services (CHF, NHIF, P4P, User Fees etc.)	Community sensitization on CHF at 22 wards by June 2013	Per diem – domestic	Can	1.0	990,000.00	Number of household enrolled
		Fuel	Person	1.0	440,000.00	
Proper disposal of hazardous waste, solid and liquid waste	To procure waste handling material for Mlali Health Centre by June 2013	Cleaning supplies	Person	1.0	1,000,000.00	Number of waste handling materials procured
TB DOTS Plus (TB HIV, MDR – TB)	To sensitize community on TB through commemoration of world TB day by June 2013	Extra-duty	Person	1.0	250,000.00	Number of meetings conducted
		Food and refreshment	Person	1.0	125,000.00	
		entertainment	Lump sum	1.0	200,000.00	
		Fuel	Liters	1.0	100,000.00	
Integrated management of childhood illnesses (IMCI)	To conduct mentoring of IMCI to 80 health staff in 30 HFs by June 2013	Special needs material and supplies	Person days	1.0	1,260,000.00	Number of health staff mentored
Provision of safe clean water hygiene and sanitation	To procure waste handling materials at 54 HFs by June 2013	Water and waste disposal	Quarter	1.0	5,928,300.00	Number of waste handling materials procured

*Source*: Kongwa District Council – Performance Budget Framework (MTEF) 2012/13.

Several respondents indicated that because of the lack of financial motivation, village health committees are virtually dormant. One member of the committee aptly conveyed a strong message on the inactivity of the village health committee in handling issues of sanitation:

Yes, the village health committee exists, but we do not do any activity unless there is an outbreak. We work under difficult conditions; we need allowances. (KI-3, Kongwa)

So the little money is spent by the district health department itself to support community action – like the distribution of mosquito nets and prophylactic drugs for neglected tropical diseases (e.g. elephantiasis). One of the key CHMT members said:

Community health activities take at most about 2% to 5% of the CCHP [council comprehensive health plan] budget and still you can find that the activities do not aim straight at empowering communities to address local needs. It [the community] is just invited to take part in the implementation. The implementer of the activities is the health department. (KI-2, Kongwa)

The observation from the above quote that the little funds do not support community-led disease prevention and health promotion activities is further corroborated by the information in the performance budget framework for Kongwa district for the year 2012–13 – as indicated in Table 4. The key message in this table with respect to resource allocation is that although the interventions appear to be broad (for instance, provision of safe clean water hygiene and sanitation) the corresponding activities are narrowly focused on strengthening capacity of health facilities (procuring waste handling materials for 54 health facilities). Little or no efforts are directed at empowering the community to carry out disease prevention and health promotion activities.

The finding that the little funds do not tangibly reach down to the community was corroborated by one of the ward health officers, who complained about the lack of financial support for preventive activities at the ward and village levels. He said:

The Ward Health Committee doesn't have any funding for implementing its activities. I do not have a means of transport. So when I have to go and meet the village health attendants I use my own money. Another challenge is that you find a villager telling you he has dug a pit for a latrine but does not have a slab; that's money. So these are the challenges. (KI-5, Kongwa)

### Local governance in disease prevention and health promotion

The governance function with respect to prevention and health promotion was examined from the perspectives of government authorities at the ward and village levels and that of community participation. The results in the following sections are presented in that order.

#### Ill-preparedness of the ward and village authorities

The respondents revealed that governance structures for prevention and health promotion at the village level are weak. We are particularly referring to the village health committee, dispensary governing committee, and ward development committee (WDC). One of the district officials was asked to comment on the role of the WDC. His response was:

Primarily the WDC support all development activities in the ward, but I can say that the WDC members do not know well their responsibilities despite the fact that we trained them; still they do not perform as expected. (KI-11, Kongwa)

It was also reported that the lack of transparency in the way the committees are constituted results in having incompetent structures:

It is true that some committees are not competent and sometimes others are elected on political bias. You know, we can go there to carry out elections, we tell the village executive officer to follow the laid-down directives, to advertise to the villagers, but for the sake of their interest the village chairman can select a person he wants. You see, then accountability will not be there. (KI-8, Kongwa)

Members of the WDC and village health committee who were interviewed admitted that they lack knowledge and skills in planning and supervising community health programs. Those limitations notwithstanding, it was reported that WDCs and village health committees have been instrumental in mobilizing people toward becoming members of the community health fund – a voluntary pre-payment scheme that offers a client (household) the opportunity to acquire a ‘health card’ after paying a contribution.

Furthermore, the committees were reported to exercise government powers in the control of cholera epidemics that used to occur in Kongwa in the recent past. One of the members of the village health committee aptly said:

When a cholera outbreak occurs our committee works in close collaboration with the village government in making sure that the rules regulating issues like the use of toilets are followed. (KI-7, Kongwa)

#### Limited community participation

Respondents expressed concern that there is limited involvement of stakeholders at the community level in planning for prevention and health promotion activities. There is insufficient participation of villagers in defining local problems requiring prevention and health promotion interventions. The existing ‘participatory planning’, in which the communities are presumed to take part through health facility governing committees, is limited in scope. In practice, as its name suggests, a health facility governing committee serves the purpose of appraising and approving ‘curative’ plans drafted by the health facility medical staff. This was well summarized by one respondent when answering a question on the role of the committee in health planning:

We receive a health plan from the doctor, and we add certain views about this and that; we discuss together until we agree that this item can be done this period and the other(s) one can be done later. (KI-6, Kongwa)

It was further reported that platforms for tapping people's felt health needs and for organizing community-based initiatives are virtually nonexistent. A few attempts made by NGOs led to the formation of some groups, but they could not be sustained beyond the lifespan of the NGOs’ projects. One respondent had this to say regarding the existence of social groups that may organize the communities toward designing and implementing community-based health programs:

Aaah, there were some groups which were involved in HIV/AIDS for example at Kibaigwa; they were groups organized by NGOs but after the NGOs had elapsed [project ended] the groups also dispersed so we do **NOT** have any group now. (KI-10, Kongwa)

Notwithstanding the above-presented shortfalls in participation, it is worth noting what existing structures do in the area of prevention and health promotion. It is prudent to acknowledge the role of village health workers (VHWs). The VHWs, normally two per village or hamlet, are people who are selected by fellow villagers through their government. Their role is to work in collaboration with a primary-level health facility (dispensary/health center) to facilitate the implementation of prevention and health promotion interventions. They do so through sensitization of community members and through taking part themselves in administering such interventions – which include, but are not limited to, the distribution of mosquito nets, weighing of children under age 5, and vitamin A supplementation. One of the VHWs summarized what they do:

We follow up the mother's and the child's health. We visit households in the hamlets on a weekly basis to follow up to ensure that all children are sent to the clinic, and if we find a sick child we advise parents to take him/her to the health facility. We use special visit forms to fill in when we visit the hamlet. We weigh children, administer vitamin A supplements for children and birth control tablets for women and educate them how to use them. (KI-9, Kongwa)

## Discussion

In this section, we discuss the key findings of the study, placing them in a wider context of existing knowledge. The study has highlighted several barriers and some opportunities regarding the implementation of disease prevention and health promotion activities at the community level in a decentralized health system. The barriers identified are: decision-makers at the national and district levels lack the necessary political will in prioritizing prevention and health promotion; the gravity of prevention and health promotion stated in the national health policy is not reflected in the district health plans; gross underfunding of community-level disease prevention and health promotion activities; and limited community participation. These shortfalls notwithstanding, important opportunities for improving the situation have been highlighted – the existence of the National Health Policy that states the importance of prevention and health promotion toward having a healthy population; the decentralized health system structure; and the existence of structures that serve as a bridge between the health system and communities.

### Ideology framing approaches to disease prevention and health promotion

The study found that respondents pointed out both national- and district-level decision-makers’ lack of political will in prioritizing prevention and health promotion activities. This might be attributed to the impact that ideological change has had on the mindsets of health system managers. In the Ujamaa ideology, the health for all strategy under the framework of PHC was at the top of the agenda of the Ministry of Health (36). During this period universal access to health care was the goal, and to that end the government considered health to be a social right rather than a matter of individual effort to seek and achieve health.

Thus, health services were provided free of charge and prevention and health promotion activities were given considerable attention (36). However, in the early 1990s changes started in the health sector and they were almost parallel to what was going on in the political sphere and reflected the influence of political ideology.

Similar experiences have been documented in Venezuela and Brazil (5). The difference between health sector reforms in Tanzania and those in Venezuela is that in the former (Tanzania) the reforms were, and still are, pushing the country to the right – paying less attention to the plight of the poor because of the introduction of user fees (37) and increasingly paying less attention to disease prevention and health promotion at the community level. On the other hand, in Venezuela, the reforms were a reaction against the poor health conditions of the poor under neoliberal policies. Armada et al. (7) report that by the late 1990s, class-based health disparities were vast. Hence, when Hugo Chavez became president, he spent his first term, between 1998 and 2002, implementing a variety of strategies to eliminate barriers to health care. These included measures such as the implementation of integrated health care, and a focus on PHC centers and on prevention activities, thus shifting the emphasis away from curative care. Similarly, in Brazil the changes in the health system occurred toward universality (5). The reforms were as a result of mobilization in health, struggling for better and inclusive health services. These struggles from several groups – trade unions, health workers, academics, and other civic groups – constituted a health movement that stirred reforms and culminated in a health system that is more democratic and put prevention at the top of the agenda. Inspired by Brazil, South Africa's Health Ministry is reforming post-apartheid a curative-oriented health system – engaging more in what has been referred to as re-engineering of PHC (29).

Moreover, the study has found that prevention and promotion activities are not adequately articulated in the comprehensive district health plan. This implies that the emphasis on prevention is merely rhetorical. Most activities targeting major diseases such as malaria, TB, and diarrhea are curative – presented as ‘case management’. Consequently, people, particularly the poor, will continue being trapped in the vicious circle of ill health. A similar observation was made by Baum et al. (6) as they argued that the health care sector in many poor countries is dominated by a Western biomedical imagination that results in curative medicine being privileged over strategies that emphasize disease prevention and health promotion. In addition, the study findings also show that the local government has delegated its responsibility to provide environmental sanitation services to the private sector and civil society organizations. Although this is an opportunity for a public–private partnership (PPP) in service provision, in a poor country a PPP strategy presents a risk of having a larger part of the population unattended to. This is because a substantial number of people may not be able to afford to pay private providers, thereby failing to dispose of waste properly. Hickel (38) attributes this pattern of environmental health management to the current political discourse of market individualism, which treats sanitation as a matter of individual achievement rather than a social right to be fulfilled by the state.

### Underfunding: a major challenge

The study has indicated that prevention and health promotion activities are grossly underfunded. From the resource allocation point of view such activities at the community level are marginalized as only 2–5% of district health funds are allocated for them. As a consequence, this small budget is spent on narrowly focused activities. For example, although the intervention is stated as ‘provision of safe clean water, hygiene, and sanitation’, the corresponding implementation activity is: procurement of waste handling materials for 54 health facilities. This pattern of expenditure leaves broader health promotion and disease prevention needs of community members unattended to. That is, the budget in Table 4 makes no mention of support for waste disposal, sanitation, and/or water safety at the household level.

As discussed in the preceding sections, underfunding reflects an inclination toward prioritizing curative over preventive intervention. This pattern of funding has been reported elsewhere (39, 40). It is surprising that this is happening in a poor country that is experiencing a double burden of disease (2). Prominent scientists in the developed world, like the Englishman Thomas McKeown, noted more than three decades ago that major contributions to improved health in those countries over the previous two centuries came more from changes in sanitary conditions than from medical interventions (41). The need to invest in improving living conditions for people for better health has been well consolidated in the report of the Commission on Social Determinants of Health (42).

In spite of this evidence, proponents of the new public health paradigm (43, 44) express their disappointment that poor countries’ financial resources for health are increasingly being spent on the provision of curative health services, especially hospital services, rather than being invested in prevention and health promotion. They add that even when health promotion activities are funded, the focus is generally on changing the behavior of individuals rather than creating wider physical, social, and economic environments supportive of healthy behavior. This is shown by Tanzania's primary health services development program (PHSDP) 2007 – 2017. It is worth highlighting that the PHSDP total budget is Tshs 11.8 trillion, but out of this only 7% has been allocated for health promotion activities, and issues of environmental sanitation are completely left out (27).

### Local governance and participation: opportunities for the future?

The study has discovered that local government structures are not well prepared in handling community prevention and health promotion activities. This deficiency may compromise their effectiveness and efficiency in providing political leadership for community-based health programs. The role of governance or political commitment in determining the success or failure of health programs has been documented elsewhere in sub-Saharan Africa (45, 46). Also the objectives stated by Saachy et al. (29) are defined for the national level and are not in themselves operational for implementation. *Accessible*, *equitable*, *and good quality health services* are not all possible to achieve to the same extent in any health system rich or even worse if poor. Such fallacy creates disagreements throughout the system and leaves the districts, facilities, and communities without procedural guidance to make ends meet. Are the district plans in essence top down frameworks to get budgets approved rather than flexible tools for the necessary local compromises? The focus should therefore rightly shift for plans to be both responsive to community demands and based on the principle of inter-sectoral collaboration (29).

In addition, ill-preparedness probably reflects the lack of health promotion policy that would have clearly prescribed an institutional framework for the organization and coordination of prevention and health promotion programs at the national, district, and community levels. The policy would clearly indicate roles of stakeholders at various levels of governance. Tanzania may learn from Nigeria, which has a health promotion policy, apart from the National Health Policy (47).

The study has found that there is limited involvement from stakeholders at the community level in planning for prevention and health promotion activities. Although health facility governing committees and VHWs serve as a bridge between the health system and the communities, their involvement is largely in approving facility-based plans. That is, the system of involving communities through committees does not provide enough room to reflect community felt health needs in the CCHP. It is therefore not surprising that community-based environmental sanitation activities are scantly budgeted for or missed out sometimes. Probably, the situation would have been different if ‘participation’ were expanded to include taking on board the views of social groups (NGOs, community-based organizations, and civil society organizations) that are interested in prevention and health promotion. This means communities need to be encouraged to form such groups where they don't exist. Such social movements could also mobilize efforts from community members themselves toward making their residences healthy. The role of social movements in bringing about better health conditions has been documented in the Kerala state of India (48) and in Brazil (5). A stronger community participation is called for now as low-income countries contemplate revitalizing the PHC approach (49).

### Study limitation

Because the study used case study design it lacked representativeness and thus findings cannot be generalized. However, being a qualitative study, its goal was not to generalize but rather to provide rich information – using multiple sources of data on barriers and opportunities for instituting disease prevention and health promotion activities at the community level in the context of health sector reforms. In addition, it would have been beneficial to have more than one case. However, this case gives indication of the barriers faced at a district level.

## Conclusion

In this era, when Tanzania is burdened with both communicable and NCDs, prevention and health promotion should be at the top of the agenda. This paper has discussed barriers to the realization of this wish – ranging from the adoption of neoliberal ideology that promotes a biomedical conception of health to limited social participation. Those gaps notwithstanding, the paper has highlighted that change is possible. A decentralized health system and existing community structures can be capitalized on. Despite operating in a neoliberal climate, a stronger role of the state is called for. That is, the state should demonstrate a clearer responsibility for ensuring community-directed health promotion supported by the health facility and district health services and other relevant sectors in implementation level collaboration. This may need both a further democratization at health facility and community levels and facilitation through stronger leadership from the state. Similar recommendations are emerging from other recent studies in Tanzania (10, 50).

Furthermore, there is a need for a national health promotion policy that will clearly chart out how multisectoral collaboration can be put into practice. The Prime Minister's office may play a stewardship role toward that direction. More importantly, the time is ripe for the evolution of social movements to demand the fulfillment of people's right to comprehensive health care provision at the community level.
